# A person-centered approach to home and community-based services outcome measurement

**DOI:** 10.3389/fresc.2023.1056530

**Published:** 2023-02-01

**Authors:** Matthew A. Roberts, Brian H. Abery

**Affiliations:** Institute on Community Integration, Rehabilitation Research and Training Center on HCBS Outcome Measurement, University of Minnesota, Minneapolis, United States

**Keywords:** HCBS, outcome measurement, measure development, person-centered analysis/approach, person-reported outcome, home and community-based services, person-centered measurement

## Abstract

In the United States, over 2.5 million people with disabilities are recipients of supports through the Center for Medicare and Medicaid Services (CMS) Home and Community-Based Services (HCBS) program. Recent decades have seen a growing focus on providing HCBS in a person-centered manner thereby supporting outcomes that are both important *for* and *to* the person. HCBS outcome measurement, however, has not kept pace with advancements in person-centered thinking as it relates to providing supports to people with disabilities. The concept of person-centered outcome measurement has been inadequately defined and is frequently misunderstood including by those in the measurement field. The authors first operationally define person-centered measurement and establish its importance within the context of HCBS and the recent CMS's Final Settings Rule. The important role that person-centered measurement has to play in quality improvement efforts in this area is then explored. A discussion is subsequently provided as to the challenges that are faced in person-centered measurement specific to the disability field. In addition to further conceptualizing and defining this form of measurement, recommendations are provided for moving the field forward.

## Introduction

Over 2.5 million people with disabilities in the United States receive Home and Community-Based Services (HCBS) through the Centers for Medicare and Medicaid Services (CMS) Long-Term Services and Supports (LTSS) program (1). The program is intended to support people with disabilities living in more inclusive settings that offer greater access to and integration within the community. Enrollment and HCBS program spending has increased significantly over recent decades as more people with disabilities prefer receiving support in their community in place of institutional settings (1). Unfortunately, in far too many cases HCBS results in a person living physically within the community but failing to experience being part of the community in a psychological and social sense [e.g. (2–4),].

The Medicaid program, including HCBS is jointly funded by the federal government and states. The federal government provides approximately fifty-six cents for every Medicaid dollar spent with states providing the balance (5). Because states are required to partially fund HCBS, they are allowed a significant amount of flexibility in how they design and administer HCBS programs. This includes the capacity to determine, (1) *who* to cover (i.e., target populations, eligibility criteria), (2) *what* to cover (a variety of waiver benefit packages), (3) *how services are delivered* (e.g., self-directed supports, through Managed Care Organizations (MCOs), and (4) *how providers are reimbursed*. Unlike Medicaid, HCBS waivers allow states to both choose and limit how many people are served under each waiver and which services are covered for which populations. Although HCBS programs vary significantly between states, some of the most common populations eligible for services include people with intellectual and developmental disabilities (IDD), physical disabilities (PD), psychiatric disabilities (psychD), traumatic and acquired brain injury (TBI/ABI), and age-related disabilities (ARD). The ability of states to limit HCBS waiver enrollment has resulted in extremely long waiting lists in most of the country because the number of people seeking services far exceeds the number of waiver slots available (6). Home and Community-Based Services are further complicated because of the extremely diverse and varied support needs of HCBS recipients. Supports range from those that are limited to periods of transition or a single context to services needed on a continuous basis. This has led states to develop and implement a wide variety of programs, services, and supports, aimed at meeting the unique support and service needs of the populations served.

As a result of the variety of programs and diversity of recipients, measurement of the quality of supports that the recipients of HCBS receive and the outcomes these individuals experience is far from a simple process. A nuanced approach needs to be taken that is responsive to a wide variety of personal and contextual factors. This process needs to be decidedly different than that currently used in medical/healthcare contexts due to the dissimilarities in the constructs measured. Unlike many outcome measures related to health (e.g., the number of urinary tract infections or falls experienced by a person, blood pressure, etc.) outcomes associated with HCBS (e.g., the extent to which people with disabilities experience a sense of social connectedness) are both more complex and difficult to assess. A second set of critical contextual factors for which one needs to account are the policies and regulations under which HCBS is implemented which vary significantly between states in the U.S.

In 2014 CMS published the Final Settings Rule for HCBS; thereby, establishing a set of requirements for the qualities that needed to be in place for HCBS settings to be eligible for reimbursement through CMS under sections 1915(c), 1915(i) and 1915(k). The provisions established an outcome-oriented definition of home and community-based services that firmly supports the self-determination and choice of recipients. Through their emphasis on person-centered services and supports, as well as service plans developed through a person-centered approach, the regulations require that planning processes reflect individually identified needs, goals, and preferences. Additionally, it strongly supports the achievement of the unique desired life outcomes of each HCBS recipient. Since its initial publication eight-years ago, states have been granted a number of extensions with respect to the date when they are required to be in compliance with the Final Settings rule. At this time, the deadline has been established as March 17, 2023. After this point, federal reimbursement for HCBS providers will be contingent on their compliance with the Settings Rule and the provision of services in a truly person-centered manner.

Long before implementation of the Final Settings Rule (7), the focus of home and community-based services had begun to move away from custodial-like care to the provision of supports that reflect the uniqueness and desired life outcomes of the recipients of support. No longer is it sufficient to focus services on what's important *for* the person. Rather, supports must reflect *both* what is important *for* and what is important *to* the person (8). For decades, states and providers have been increasing efforts to design services to be more person-centered. The State of Minnesota (U.S.) Department of Human Services, for example, has over the past 5-years funded a program of “person-centered thinking and planning” education for HCBS providers from across the state. The goal of this program is to equip provider agencies with the knowledge and tools necessary to plan and provide services in a person-centered manner while meeting the requirements of the Final Settings Rule.

In addition to the CMS/HCBS system's move toward person-centered service provision, there are legal and compliance motivations within the HCBS environment that support the need for measurement that is person-centered. In 1999 the U.S. Supreme Court ruled in *Olmstead v. L.C.* that unjustified segregation of persons with disabilities constituted discrimination and was in direct violation of title II of the Americans with Disabilities Act. Under the Olmstead decision (9), states in the U.S. are now obligated to provide services for people with disabilities in the most inclusive community settings possible as well as support them to achieve desired life outcomes. In many states, obligations under the Olmstead decision continue to be monitored by court-appointed staff to ensure that progress is being made with respect to outcomes. To fully measure the effectiveness of programs that provide services and supports in meeting Olmstead requirements, a person-centered approach to measurement is needed. The approach needs to emphasize the degree to which the outcomes experienced by HCBS recipients match their needs and preferences and move them forward in achieving desired life outcomes.

This article is intended to correct misconceptions that many professionals in community living have about person-centered measurement, discuss the need for a person-centered approach to measurement in this area, and review the strengths and limitations of existing as well as person-centered approaches to measurement in the field of home and community-based services. Information regarding the development process that staff at the RTC/OM are using is provided to supplement the discussion and provide readers with a general idea as to a process that could be used to move in this direction.

## The need for new approaches to HCBS outcome measurement

There has been great interest in assessing the outcomes of HCBS recipients over the past twenty-years. During this period, The Human Services Research Institute's *National Core Indicators*, the Center for Quality Leadership's *Personal Outcome Measures*, and the CAHPS Home and Community-Based Services Survey have all been developed and are being used by both states and support providers as one means through which to demonstrate compliance with CMS regulations. Each of these approaches has its strengths. However, all have significant limitations that lead to the need to develop new measurement approaches that address these shortcomings.

The *National Core Indicators (NCI and NCI-AD)* is currently the most widely used tool in the U.S. for the assessment of outcomes associated with the receipt of home and community-based services. The instrument was developed and validated as a state-level compliance measure and not intended to be used at the provider or individual level for quality improvement, service plan development, and/or outcome assessment. Although the NCI includes indicators in a variety of areas, it is intended to be administered (and was validated) at the instrument level as opposed to on an indicator-by-indicator basis. Users are therefore required to administer items related to all indicators as opposed to only those in which there is a specific interest. It should also be noted that although NCI and NCI-AD have been used with populations beyond those for which they were intended (i.e., people with IDD, physical, and age-related disabilities) these tools have only been validated for use with the limited disability groups noted. In addition, research has indicated that while some NCI indicators hold together well psychometrically (10), others do not (11).

CQL's Personal Outcome Measures (12) is one of the better-developed and validated HCBS Outcome tools. It is part of a commercially available system of assessment that can be used to support provider quality improvement efforts. It has been validated with a much wider variety of people with disabilities than the NCI and possesses good psychometric properties. The instrument is administered in a conversational manner to people with disabilities and includes 171 required items. A manual describing administrative procedures and items is accessible online with training available both in-person and online for organizations interested in using this tool.

A third approach to outcome assessment in the human services field that has recently been championed by the Center for Medicaid and Medicare Services is the *HCBS CAHPS Survey*. This CAHPS is a questionnaire with 69 core items developed for measuring the experiences of people with disabilities who are HCBS recipients. The CAHPS, unfortunately, currently has limited data available with respect to its validity or reliability. Internal consistency reliabilities for seventeen of its nineteen measures fail to meet even the most basic criteria for psychometric acceptability, there are serious questions about the representativeness of the sample used for the field study as well as the evidence presented to support validity, and in a number of indicator areas, there appears to be a ceiling effect with the overwhelming majority of respondents indicating the highest possible level of service quality or personal outcomes (13).

In addition to the individual shortcomings of the most widely used HCBS outcome measures, there are three additional limitations that cut across the instruments noted above as well as other outcome assessment tools that contribute to the need for development of new measurement approaches. The first of these entails the relatively small percentage of items included in most HCBS outcome measurement instruments that meet the criteria for person-centeredness. A recent study of 140 outcome measures used with HCBS populations (14) found that only 36% of the items included in these tools were person-centered in nature. Although some outcome measures (e.g., the CQL-POM) are more person-centered than others, the overall results of this study clearly indicate the need for approaches to assessment that place greater priority of assessing outcomes within the context of what is most important to individual persons with disabilities. Overall, measurement of the extent to which HCBS recipients experience *person-centered outcomes*—outcomes that go beyond compliance and include assessment of what is important to the person, has lagged far behind the push for person-centered services. Providing person-centered services, however, is incompatible with measurement that does not consider an individual's desired life outcomes.

A second shortcoming that cuts across tools is the lack of evidence that they are sufficiently sensitive to change over time that they can be used in a longitudinal manner. Some developers, such as HSRI (NCI) explicitly state that their measures are not intended to be used longitudinally. Others (e.g., CQL, CAHPS) have yet to provide evidence that, when used in a longitudinal manner, their measures are sufficiently sensitive to change that they can be used as evidence of the effectiveness/efficacy of quality improvement efforts or changes that take place in a HCBS recipient's life. A third reason to think about the development of new approaches to outcome measurement in HCBS emanates from the resources needed to administer measures at a time when the human services field is experiencing serious workforce shortages. All of the tools referenced above are intended to be administered in their entirety as full instruments. They are neither modular in format allowing for administration focused on only one or a few indicators, nor tiered and able to provide both a quick general overview of indicators as well as a more in-depth assessment.

The Rehabilitation Research and Training Center on HCBS Outcome Measurement (RTC/OM) at the University of Minnesota, funded by the National Institute on Disability, Independent Living, and Rehabilitation Research (NIDILRR), was created for the purpose of improving HCBS outcome measurement in the United States. The center has conducted its work in multiple phases beginning with the selection and conceptualization of several measurement domains for the development of person-centered measures and, in later phases, the testing and validation of those measures. A key part of the process in completing the RTC/OM phases has been defining person-centered measurement and executing a process for developing and validating person-centered measurement tools.

Person-centered HCBS outcome measurement tools are essential for acquiring valid information regarding both the extent to which the services provided to people with disabilities are truly person-centered *and* the extent to which these supports foster the achievement of person-centered goals. When this form of measurement is not used, the information collected yields data solely with respect to the extent to which the person's experiences are aligned with benchmarks defined by someone other than the individual with a disability. This form of “non-person-centered measurement” and the benchmarks on which it is based assumes that, unlike the general population, all people with disabilities desire the same life outcomes with respect to employment, education, housing, and social relationships. This assumption must be argued to hold regardless of differences in the cultural, racial/ethnic, and gender make-up of the people in question as well as variation in the types of disability they experience or their level of support needs. Previous research, however, suggests that these assumptions are not supported and that future aspirations as well as how people define their quality of life are as varied as within the general population (15–18). A number of researchers (16, 19–21) therefore suggested some time ago that tools need to be developed that rely less on generalized outcome measures and consider both the unique profiles of people with disabilities and the social and environmental factors that influence the outcomes they both desire and experience.

Much of today's focus on outcome measurement is driven by the need of program administrators and federal and state agencies to have evidence of the impact of HCBS on the outcomes that the recipients of services experience. This is to both demonstrate compliance with current regulations and support continued congressional funding of HCBS programs. Given the Final Settings Rule (7) it is critical to collect and share data with funding agencies that demonstrate the extent to which HCBS are supporting person-centered outcomes associated with the full inclusion in the community. It is also essential that measures are available to track how policy changes, as well as efforts at quality improvement, assess the extent to which services are provided in the manner intended (e.g., program fidelity) and produce better outcomes. In the remainder of this article, we explore the concept of person-centered measurement within the context of HCBS, how it can be operationalized, the challenges with using this approach, and strategies that can be used to develop measures that achieve this measurement pre-requisite.

## The concept of person-centeredness

The concept of person-centeredness has existed for decades and can be traced back to Carl Rogers [e.g., (22, 23)]. Fundamentally, person-centeredness posits that the person has the greatest understanding of themselves, and a full appreciation and involvement of the person is necessary to achieve successful outcomes (23). Over the past forty-years the field of disability services has evolved to include person-centeredness in the areas of planning, service delivery and coordination, outcomes, and assessment. Person-centered practice emerged in the United States during the early 1980s as people with IDD transitioned from institutional to community-living. With this came the need for individualized service plans to fit the needs of each person living within the community based upon their preferences and desired life outcomes (24, 25). Recent decades have also seen the parallel development of patient-centered models in health care [e.g., (26–28)]. CMS has pivoted to support the incorporation of person-centered planning and practices into disability support systems (i.e., HCBS, LTSS) including recent efforts by a national stakeholder committee to further define person-centered planning and practices, generate core service delivery competencies in the area, and develop compatible measurement frameworks (29). As the sophistication of person-centered practices have increased, even more attention is needed toward measuring whether the services result in the outcomes that are important to individuals.

Person-centeredness as we define it is essential to treating people with disabilities with fairness and equity. The Convention on the Rights of Persons with Disabilities (30) asserts the right of every person with a disability to live and enjoy their life on an “equal basis with others” (article 10) as fully included participants in society. This includes the right to personal self-determination. In the United States, the HCBS Settings Rule (7) requires that people with disabilities receives support and services that are provided in ways that are based on their personal preferences and assist them in achieving desired life outcomes. This includes the right to choose where one will live and with whom; if and in what type of job one will work, as well as the types and limits of the supports one receives. Environments and the professionals providing supports must promote the individuals having control over day-to-day choices including the kinds of support they will receive (31). The purpose of this rule is to ensure HCBS are provided in a manner that promotes both community inclusion and self-determination and is delivered based on what is important to each individual, rather than asking people to adapt or compromise based on what is most convenient or available within the system. This focus on ensuring that each person has opportunities to make meaningful choices about support to be received and about his/her life, is in keeping with both the CRPD (30) and the rallying call of people with disabilities who have for years been stating, “nothing about us, without us.”

The changes noted above are grounded in a paradigm shift in the field of disability services from a *medical model*, which focuses on somehow changing or “fixing” people with disabilities so that they will better fit into the existing society, to a *social-ecological model* of disability, which shifts the onus to creating environments that best accommodate people with disabilities with the intent that they experience life as full members of the community. This paradigm shift requires that HCBS be individualized to address what is important both *to* and *for* each person with a disability, rather than designing service systems that assume that all people with disabilities desire to experience the same or similar life outcomes. This paradigm shift demands that tools designed to measure the effectiveness or quality of HCBS must be person-centered and based on the needs and preferences of each individual serving as the benchmark to which we compare experienced outcomes. For example, the idea that all people with disabilities desire to have a large number of friends and that more friends is a *better* outcome than fewer friends may reflect the preferences of some but certainly not all, people with as well as without disabilities. Some people may feel socially connected to their communities if they have a few close friends. For others, however, a larger number of social relationships will be necessary. Person-centeredness is paramount to ensure both equality and equity with respect to outcomes whether one is considering national or international policy and regulations.

Delivering supports in a person-centered manner requires a responsive service system. It changes the way services are delivered from a top-down approach in which the consumer receives supports according to parameters defined by state and federal agencies funding those supports, to a more bottom-up approach in which the parameters are more flexible and based on the individual needs and preferences of people with disabilities. This process begins with people with disabilities effectively communicating their desired life outcomes and subsequently advocating for supports designed to help them achieve these ends. At a second level, it entails staff or caregivers who directly work with the recipients of services and understand their needs and desires ensuring that service plans and day-to-day supports are directed at facilitating people with disabilities achieving these outcomes. Beyond this, it extends to the leadership of service provider's and the extent to which they support and enable staff to provide person-centered supports. At the uppermost level it extends to government systems that regulate and fund the provider agencies. A similar paradigm shift has taken place in the United States educational system in which there has been a move away from schools dictating educational plans for students with disabilities in favor of the student, family, and school becoming partners in creating an educational plan and determining the best supports for the student.

The concept of person-centered supports is not focused on each person experiencing every outcome they desire. Rather, it focuses on the extent to which a person's desired life outcomes are heard and acknowledged by their planning team, included in their service plan, and efforts made to make progress toward them. Making supports truly person-centered also requires ongoing assessment of the support recipient's preferences, personal goals, needs, and progress/outcomes since these are likely to change over time, as well as a willingness at the provider level to change policies when necessary and adjust services to support the individual in pursuit of their personal goals.

## Person-centered measurement

Despite decades of research defining the person-centered concepts, the concept of *person-centered measurement* has not been well defined or understood. Consequently, measure developers have struggled with identifying exactly what makes a measure or item person-centered. Historically, at least in western cultures, measurement has focused on comparing the performance or experiences of a target person to benchmarks or the performance or outcomes experienced by other people (i.e., the norming group). This approach makes sense and works well when one is measuring outcomes against which there is a known performance criteria or standard that one desires to see a person attain (a benchmark) or it is important to determine an individual's performance relative to a larger group (norm-referenced). For example, in the healthcare field, person-centered practice frameworks [e.g., (27, 28, 32)] have established standards that can be used to compare achieved outcomes against.

In some cases, however, there are no real standards against which to measure a person's outcomes or performance other than the extent to which they meet the individual's desired outcomes and personal needs. We argue that this is the case when one's focus is on measuring the outcomes people with disabilities who receive home and community-based services experience. In these instances, the “standard” against which to compare outcomes or experiences needs to be based on the service recipient's personally defined preferences or goals—not those that other people or the service system sets for them.

In the context of measuring outcomes associated with people who receive HCBS, we contend that for measures to truly be person-centered they meet a number of specific criteria. This is not intended to imply that all HCBS quality measures need to be person-centered. For example, some indicators of workforce characteristics would not make sense to design in a person-centered manner. Rather, we believe that attention in this area needs to be focused on measures of the personal outcomes that people with disabilities desire to experience when they are recipients of HCBS.

From their conceptualization, person-centered HCBS outcome measures need to be designed with the intent that they will be responded to by people with disabilities themselves. Although informed respondents can often provide valuable information with respect to another person's experiences, being able to accurately articulate what another individual believes they need, outcomes they desire to experience, and/or the degree to which they view themselves as making progress toward achieving those outcomes is a difficult task. Previous research indicates that most people have a difficult time understanding how others experience their world, what they desire, or when they are satisfied with the outcomes they experience [e.g., (33–37)]. Designing measures so that they can be directly responded to by people with disabilities themselves places a heavy responsibility on developers that measures are designed so that they (a) are clearly understood by the intended respondents; (b) based upon a time frame that respondent can conceptualize; (c) provide response options that accurately reflect an individual's experiences; and (d) are able to be responded to in a manner that permits people to indicate the extent to which the outcomes they are experiencing align with their desired level of the outcome or indicate progress. For example, although an item that asks a respondent how many hours per-week they work provides some useful data, asking that question and following-up with, “To what extent are you working the number of hours you desire to work?” has the potential to provide more person-centered information.

**Figure 1:**
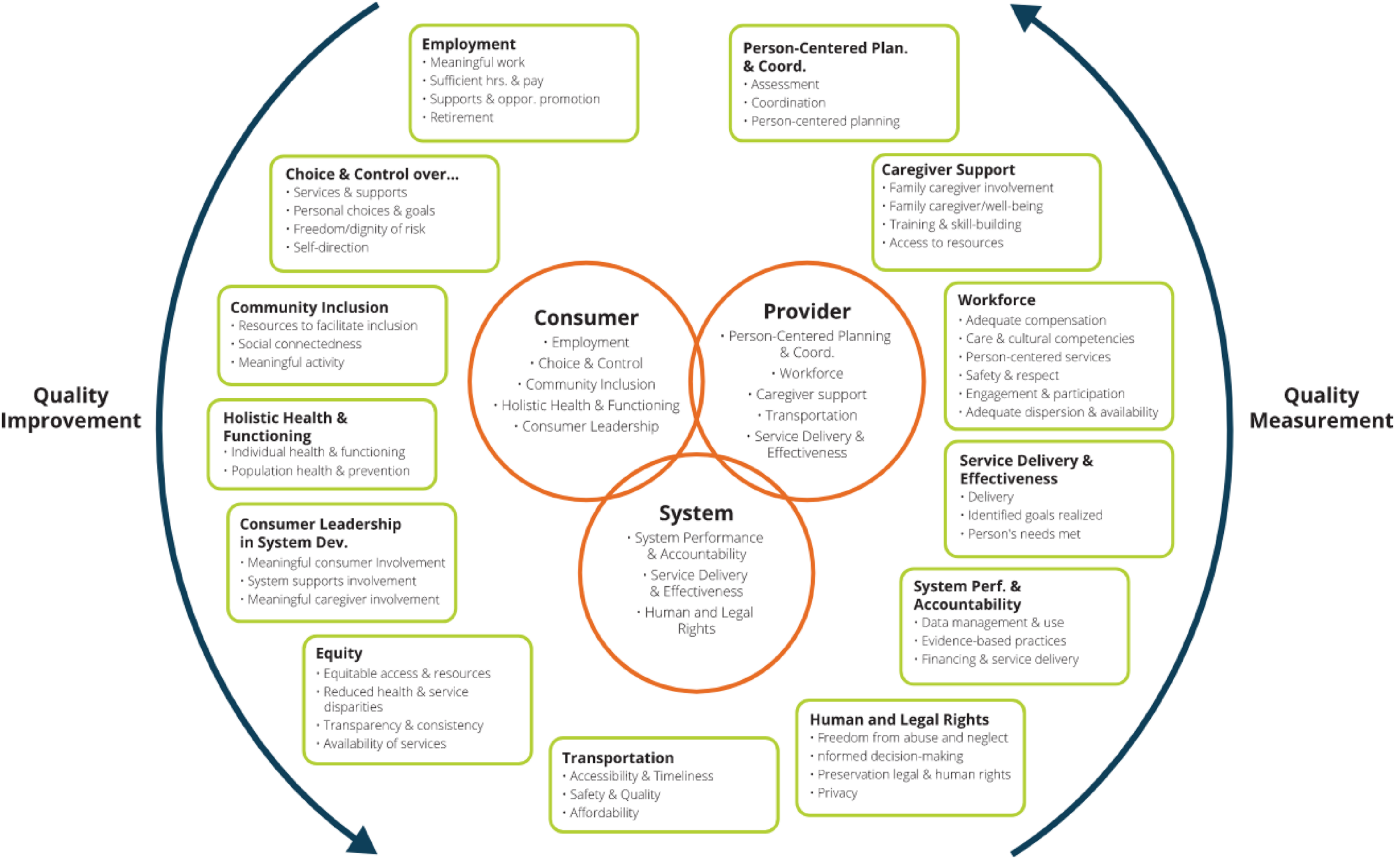
Revised national quality forum HCBS outcome measurement framework training and training center on home and community-based services outcome measurement (RTC/OM), a systematic search of HCBS outcome measures was undertaken and over 200 instruments reviewed for inclusion into a database of HCBS outcome measures. The items for each instrument were coded into the domains and subdomains included in the NQF framework and for a number of other characteristics including whether they met the criteria for being person-centered. To be coded as “person-centered” an item needed to meet two simple criteria: (a) be designed to be responded to by the person with a disability or when a direct response was impossible, a proxy; (b) enable the responding person to express a preference, desire, need, want, or the extent to which these have been met (see https://rtcom.umn.edu/database). Table 1 summarizes the percentage of items coded as “person-centered” for relevant NQF domains.

A second critical aspect of person-centered measurement is its focus on outcomes that are *both important for* and *important to* HCBS recipients. Six years ago, the National Quality Forum utilized an expert panel to develop recommendations for the inclusion and prioritization of domains to address performance measure gaps in HCBS outcome measurement (38). On the basis of their work, the NQF developed a framework of eleven core domains each of which included 4–7 subdomains reflecting HCBS quality. As part of a multi-year center, the University of Minnesota's Rehabilitation Research and Training Center on Home and Community-Based Services Outcome Measurement (RTC/OM) undertook a national validation study of the framework with stakeholders representing multiple groups including people with a variety of disabilities (IDD, PD, TBI/ABI, PsychD, Age-Related), family members, HCBS providers, and state and national level program administrators. Results indicated strong support for the framework as well as some needed refinements (39). This refined framework (see Figure 1) includes a myriad of outcomes that require measurement at a person-centered level. However, the developers of new measures as well as the majority of currently available HCBS outcome measures all too often assume that achieving desired life outcomes in all domains and subdomains are of equal importance to people receiving supports. Given differences in people's preferences and the limited resources available within the HCBS system, an approach which weights outcomes with respect to their *importance to the individual* needs to be incorporated if we are to achieve truly person-centered measurement based upon the unique needs and desired life outcomes of each individual.

**Table 1:** Percentage of items coded as person-centered by NQF domain.

NQF domain	Items coded	% PC
Choice and Control	1,144	49.7%
Community Inclusion	1,757	31.8%
Equity	86	30.2%
Holistic Health and Functioning	1,129	34.1%
Human and Legal Rights	543	23.8%
Person-Centered Planning and Coordination	524	47.3%
Service Delivery and Effectiveness	784	32.4%
*Across all Domains* ^a^	*5,275*	*34.6%*

^a^ Overall sum and percentage exclude items coded into two or more domains.

A third criteria that we believe is essential for person-centered systems of measurement are their capacity to be used in a longitudinal manner. Given that such systems would be developed with the intent to focus on measuring the extent to which HCBS recipients are progressing toward or achieving their desired life outcomes, suitability for longitudinal use and sensitivity to change over time will be critical characteristics of the system. Current approaches to HCBS outcome measurement are primarily cross-sectional (i.e., the outcomes/experiences of a different set of respondents are assessed every year). As a result, one needs to make many assumptions with respect to the year-to-year representativeness and comparability of samples in order to interpret results. As a result, interpretations of improvement/progress toward outcomes on the part of individuals are not possible and systems improvement is only able to be made in an indirect manner. Although measurement systems of this type do provide important information (e.g., compliance with state and federal regulations) they leave much to be desired as instruments of quality improvement.

Given the criteria specified, what is the extent to which measures currently used to assess the outcomes of HCBS recipients are person-centered? As part of the Rehabilitation Research.

The table indicates that the majority of items that could be coded into an HCBS outcome domain did not meet the established criteria for being person-centered. To illustrate, one recently developed tool is the CAHPS Home and Community-Based Services Survey (40). A total of 87 items were coded into various domains of the NQF framework, but only 10 of these items were coded as person-centered (41). During a follow-up effort, seven states were identified as using this tool for outcome measurement in HCBS programs (42).

In addition to people with disabilities, there are several other stakeholder groups that would benefit from the expansion of person-centered HCBS outcome measurement. Families, together with their member with a disability could use this information to better determine which provider organizations they desire to provide supports as well as to ascertain whether the services their member with a disability receives are facilitating progress toward or the achievement of desired life outcomes. Service providers would also profit from this information utilizing it to help determine if programmatic and policy changes intended to improve service quality have been successful and to assess whether supports are meeting recipients' needs. Managed Care Organizations (MCOS) which are increasingly administering HCBS in the U.S. are another stakeholder group that would benefit from access to psychometrically sound person-centered measures. Such measures would be useful for providing information about the extent to which individual providers within the MCO network are delivering services that result in person-centered outcomes and use this information to both select additional HCBS provider agencies and potentially incentivize those that are facilitating personal outcomes of HCBS recipients that are consistent with the Centers for Medicaid and Medicare Services Final Settings Rule (7).

## Person-centered measurement and goal attainment scaling

It is our belief that HCBS outcome measurement should have a prime focus on quality improvement. Therefore, the ability to track the extent to which the services HCBS recipients receive support their making progress toward achieving desired life outcomes over time is essential. This requires measures that can be used on multiple occasions with the same respondents. Such measures need to be sufficiently sensitive to change across time that they are able to detect meaningful differences in a person's experiences, outcomes, and goal attainment within relatively short periods (e.g., 6–12 months). To achieve this level of measurement, developers need to be sensitive both to the manner in which items are worded and the response options that respondents are provided. The former corresponds to developing items that are sufficiently specific so that change can be detected over short periods. The latter focuses on providing those with whom measures are being used with options for responding that allow them to indicate meaningful change in their lives. One approach that we believe responds to this need is the use of *Goal Attainment Scaling* (GAS).

Goal Attainment Scaling (GAS) is a method for writing personalized evaluation scales (43) in order to quantify progress toward defined goals. Developed over 50-years ago, this approach to measurement is attracting growing interest, especially within the context of intervention and clinical research because it permits the efficacy of treatments to be assessed with respect to goals set by the clients themselves. Although GAS was initially used primarily within the health-related rehabilitation fields for people with physical disabilities and in rehabilitation psychology (44, 45), recent years have found it increasingly used as an outcome measure for people of all ages with a wide variety of functional limitations including adults (46–52), aging adults (53, 54) children in clinical and special education settings (55–62), infants (63) and with parents of children with disabilities (64).

Most recently, Shogren and colleagues (65) have made the case for the use of Goal Attainment Scaling in research focused on determining the efficacy of interventions for people with intellectual and developmental disabilities. She and her colleagues contend that the use of this approach: (a) supports the need for valid and reliable processes to quantify the progress individuals make toward achieving or making progress toward personal goals, (b) allows for the aggregation of data across individuals to determine group effects, and (c) is consistent with the movement toward person-centered services and the support of the self-determination among people with IDD. Within the context of HCBS outcomes measurement, Shogren and her group make the case that traditionally used non-GAS measures while providing information with respect to outcomes, fail to provide a holistic representation of the degree to which the outcomes of programs are truly a function of the supports people receive and the relationship of individuals with disabilities achieving personal goals and longer-term outcomes. Several literature reviews on GAS have been published (54, 66–70) and together with studies that specifically addressed the psychometric qualities of GAS (71–74) these publications suggest that this approach has more than sufficient evidence to support its use in a wide variety of measurement contexts.

How is the process of goal attainment scaling implemented? The first step in GAS is to identify an individualized goal of interest. In the context of HCBS measurement, this could be a desired life outcome either associated with a specific domain of NQF HCBS Outcome Measurement Framework (38) or external to it. The individual with a disability him/herself, with support from others when needed, must be the person on which this phase of GAS centers. The second phase of GAS entails determining what outcomes or behavior will reflect varying degrees of goal attainment in relation to those outcomes of interest. Once again, this step needs to be driven by the perspective of the HCBS recipient as opposed to others. A third step in developing a GAS approach to outcome measurement entails the development of five-point rating scales that operationalize expected outcomes. Typically, these range from −2 (much less than expected) through 0 (expected) to +2 (much more than expected). An alternative approach that has been used entails levels ranging from a −2 (no change) to a +2 (much better than expected) outcome or attainment of the desired outcome or goal. As a next step in the process, a specific time interval for evaluation of progress needs to be set. Depending on the goals or desired outcomes in question this can be anywhere from a few weeks to a full year. The final step in the GAS process is to rate goal attainment after the specified period, using the established GAS rubric and calculate the overall attainment score for the individual's goals.

A number of diverse ways of analyzing the results of GAS can be found in the literature. Scoring each and every goal between −2 and +2 provides a direct indication of the degree to which each goal has been achieved (45). This approach is likely to be easily understood by both HCBS recipients and providers and can be used at the individual level. However, it makes it difficult to undertake aggregated statistical analysis. A second approach is to transform raw GAS scores into T-scores enabling normalization and analysis using a variety of parametric statistics. This is the approach recommended by Kiresuk (43, 75) and covered in depth by Krasny-Pacini and colleagues (44). The mean of raw scores (76) as well as the sum of the differences between the baseline and the level of goal attainment for each person's goals have also been approaches (77). The T-score is the most frequently used method allowing for the reporting of results as a single standardized value.

GAS is a valuable, but certainly not the only strategy to use in order to move toward more person-centered measurement. In some cases, those undertaking program evaluation are interested in aspects of an individual's experience unrelated to the specific goals or outcomes they set for themselves. In other contexts (e.g., freedom from abuse and neglect) personal goals are subservient to state and federal legislation that fully prohibits these negative outcomes. In these instances, ensuring that measure items meet the criteria noted above and are responded to directly by persons with disabilities, solicit a preference, desire, want, need or emotional state from respondents, and provide a chance for people to indicate the degree to which those preferences, needs, etc. are being met provide what we consider to be person-centered data.

## Challenges to using person-centered measurement and goal attainment scaling

The GAS process is highly adaptable and has great potential to be used as a person-centered outcome measure to establish the impact of supports received by HCBS recipients regardless of differences in desired outcomes or goals [see (44, 65, 78)]. It is not, however, without its challenges. As Shogren and colleagues (65) point out individualization of goals though desired in practice (79), can change the nature of goals. Goals lacking in precision also have implications for the accuracy of measurement. Shogren also points out that, in many cases, goals identified in one context (e.g., transition planning) are specific to other environments (e.g., work) increasing the challenge of meaningful and reliable GAS rating scales.

Additional challenges to using goal attainment scaling and person-centered approaches within the context of HCBS outcome measurement are related to (a) accounting for differences in the *importance* (to the person with a disability in question) of specific goals and outcomes, (b) the *challenges* a person is likely to face in making progress toward or achieving desired life outcomes, (c) the *time* it is likely to take to achieve sufficient progress to goals for change to be measurable, and (d) the *motivation* an HCBS recipient has with respect to working toward specific desired outcomes.

When attempting to measure the overall quality of outcomes a person experiences, it is critical to account for the fact that, for most people, some goals/desired life outcomes are significantly more important to achieve than others. Achieving a desired life outcome of a low level of importance is not the same as realizing one that ranks at the top of one's list of important outcomes. GAS is able address this challenge through the weighting of T-scores, giving more weight to certain outcomes/goals and their corresponding scales than others based on an individual's importance weightings. A similar situation exists with respect to the level of challenge or difficulty one is likely to encounter to achieve a specific goal. Some goals (e.g., moving from one occupation to a new one that will require extensive additional training) will require an individual to navigate significantly greater obstacles than other outcomes (e.g., acquiring a bicycle). Various weighting methods have been suggested in the literature as a function of the difficulty (80) and the probability of attaining the goal (81) that when employed properly allow one to take these factors into consideration.

A third challenge that must be confronted when using person-centered and GAS-based measures within an HCBS context relates to time. More specifically, how much time will be necessary in order for individuals to make sufficient progress toward their goals and desired outcomes so that change is detectable? This will likely vary significantly based upon the nature of a person's goals as well as the quality and specificity of the goals that have been developed. In addition, it will be affected by how organizations undertaking measurement/evaluation use the data collected. Regarding the latter, it is important to differentiate between whether the intent of measurement is as part of a formative or summative evaluation process. When the intent is the former, the idea is to monitor progress, ensure that recipients of services are on the right track to eventually achieve desired goals and make needed changes when initial strategies are not working. This approach requires goals to be specified in a fine-toothed manner and likely requires the breaking down of large/long-term goals into subgoals that are measurable over a shorter period of time. If the intent of measurement is summative, or primarily focused on the achievement of a standard after a person has been exposed to a program of supports and services, goals and the measures used to assess them are unlikely to need to be as fine-grained as those employed for assessment of a formative nature.

A final critical challenge to overcome if person-centered approaches to measurement are to be used within the context of HCBS is the necessity of obtaining responses directly from people with disabilities. Minimizing the use of administrative data sets and proxy respondents as a source of data is essential given that in many cases, the questions being asked can best or in some cases only be answered in a valid manner by the people in question themselves. HCBS recipients, however, vary greatly with respect to the nature of their disabilities, intensity of supports they need, and their capacities. This includes the ability to communicate their thoughts and feelings in a valid and reliable manner. Some individuals may not possess or may have lost the capacity for functional communication. The extent to which people who experience disabilities that have a cognitive component can provide valid self-report responses data needs to be carefully considered (82). Some individuals with intellectual and developmental disabilities, psychiatric disabilities, and TBI/ABI experience limitations with respect to understanding the meaning of questions, being able to accurately recall information, determine the order in which events took place, or make comparisons. Others may have great difficulty expressing their thoughts and feelings (83).

The language and cognitive demands of items as well as the response formats provided whether in the form of a survey or interview can present challenges to the reliability and validity of data collected. Items phrased negatively have been found more difficult for individuals with cognitive limitations to comprehend (83). Additionally, research indicates that questions about frequency, time, or abstract concepts (e.g., how inclusive do you feel when in the community?) can also be problematic (82, 84, 85). Fang and colleagues reported that complex rating scales are often quite difficult for people with cognitive limitations to comprehend (86). For some years now it has been known that people with intellectual disabilities may be prone to response bias including a tendency to select positive/agreeable response options (87–89) and both acquiescence and recency bias (84, 90) irrespective of one's true opinion). Additional research indicates that the higher the cognitive demand of a question/item, the more likely it is that a person will provide a biased response (91).

In spite of the challenges, a number of approaches have been shown to reduce the difficulties most people with intellectual disabilities experience in responding to self-report interview questions. These include tools to engage people with IDD such as participatory photographic research methods (92) or visual and metaphorical devices (93). Hollomotz (94) found that when questions were posed in plain language and accompanied by concrete reference tools, including picture cards and photo-story vignettes people with IDD were able to respond to a range of questions about sensitive topics including sex, risk, and their social and leisure lives. Cognitive and language limitations have been shown to be able to be minimized through the use of simplified question wording and response formats (84, 95, 96). Limitations in the ability of interviewees to respond to questions have been avoided through the use of response formats that require no more than a pointing response to emojis/icons or pictures. Simple response scales (e.g., yes, sometimes, no) have also been successfully used. Difficulties responding to questions regarding time have been minimized through the use of reference points with which an individual is likely to be familiar (e.g., birthdays or holidays). Adjusting the depth of questioning in line with what a respondent wants to or can offer has also been found to enhance the quality of data obtained as has a simplified conversational approach (97–100).

The strategies noted above have been shown to increase the capacity of people with a variety of disabilities to respond in a reliable and valid manner to self-report measures. There are still some individuals, however, who in spite of these approaches are unable to report accurately on the outcomes they experience. In these cases, a proxy respondent may be needed. In addition to the obvious person-centeredness limitations of not obtaining a direct response from the person, there are other difficulties associated with proxy responding that need to be considered. There may not be a proxy who truly knows the person well enough to provide a valid response. Moreover, evidence suggests that the validity of proxy responses decreases when the judgment/response made on behalf of the person is more subjective (101).

This does not mean however proxy responses do not provide a viable and important alternative when there is no other way to solicit information. For the past twenty-years, research has been undertaken in an attempt to better understand how proxy data can be used and its limitations. Stancliffe (102) and McVilly and colleagues (103) both found that in contrast to earlier research utilizing non-standardized approaches to assess quality of life, use of the Quality of Life Questionnaire (QOL-Q) resulted in a high degree of concordance between people with IDD and proxy respondents. More recently, Simões & Santos (104) as well as a host of other researchers [e.g., (105–107)] who have compared the points of view of clients with IDD and independent ratings of family members and staff, have found moderate to strong correlations (.69–.89) between persons with disabilities and knowledgeable caregivers when comparing various aspects of quality of life on both the QOL-Q and the WHOQOL-BREF. This does not mean that differences in perspectives do not exist. As might be expected, agreement is higher in some areas than others with higher levels of concordance with respect to more objective assessments of conditions of life and lower when the focus is on perceptions of satisfaction [e.g., (108)]. With respect to the latter, most findings indicate that the ratings of people with IDD are higher than those of family and staff (104, 105, 107). As Perkins (109) concluded, overall, proxy reports can be useful in determining a variety of aspects of well-being of people with disabilities as long as those using measures keep in mind that variety of factors that have the capacity to enhance (e.g., experiences/abilities that are more objectively assessed, and attention to question format) or diminish (e.g., experiences/abilities that are more subjectively assessed, severity of dementia, and level of ID) the quality of information obtained.

Alternative methods of using a proxy have also been put forth. Kaye (110) proposed that using a proxy-assisted approach can sometimes be effective as a compromise between proxy-only and self-reported responding methods. Using this method, the proxy responds with the person who has difficulty responding for themselves to assist with choosing a response. In an application of this approach to healthcare experiences, Elliot et al. (111) found a reduction in the level of bias compared to a proxy-only approach, but the proxy-assisted responses were still found to have a greater potential for bias than self-reports. Rand and colleagues (112) proposed a novel method of obtaining responses from a proxy. They posited that the proxy needed to first provide their *own* opinion on the outcome experienced by the person they are responding for prior to providing a response on how they think the person with a disability might respond. They suggest that this may reduce some of the response bias related to the proxy's own opinion. This method was utilized by the RTC/OM to develop proxy measures that include a reduced set of items that proxy respondents reported as both understandable and answerable during cognitive testing. This approach has the added benefit of providing two unique pieces of information for each item answered: (1) the opinion of someone who knows the person well and; (2) the proxy's best guess as to how the person with a disability would respond if they could. Further research is needed on this approach as to whether the proxy can sufficiently separate these distinct types of information when responding.

A final challenge to the use of person-centered measurement in the HCBS field results from the limited financial resources available to providers to undertake such evaluation and the workforce shortages endemic to the field. However, any type of assessment or progress monitoring, including alternative approaches, is going to require resources. Some (e.g., administrative data sets) might be less expensive than securing the information from persons with disabilities themselves. However, these alternatives would certainly: (a) not be person-centered and (b) be significantly less likely to provide *actionable* data that would lead to improved individual outcomes and/or enhanced supports and services. Although desired life outcomes/goals as well as their importance to an individual are in fact likely to change over time, the approach we advocate can be effectively used as a progress monitoring tool to detect these changes and allow for modifications in both supports and outcome measurement related to an individual's current desired life outcomes. In conjunction with person-centered approaches to planning, assessment, and services/supports this approach possesses the potential to more effectively ensure that the support received by an HCBS recipient actually address outcomes relevant to the lives they desire to lead. The approach for which we are advocating is broader than merely assessing the extent to which people with disabilities are making progress or achieving their goals. It includes an assessment of outcome domains and subdomains laid out in the National Quality Forum's HCBS Outcome Measurement Framework. This approach is needed by providers to assure both state and federal funding agencies that services and supports as well as the outcomes experienced by HCBS recipients and in concordance with the Final Settings Rule (7).

## The need for a person-centered measure development framework

As Lipson (113) notes, there has been a significant amount of research and development in the area of *person-reported* measurement as it relates to people with disabilities. Unfortun-ately, there appears to be a mistaken belief that *person-reported* measures are equivalent to *person-centered* measures. As noted previously, this is not the case. Both CMS and the National Quality Forum (NQF) have provided extensive guidance on measure development (see CMS MMS Blueprint, 5 & HCBS Outcome Measurement Framework, 38) in addition to guidance on developing person-reported measures. Yet, there is little guidance on how to develop measures that are person-centered.

Person-centered measurement infuses person-centeredness into the measurement tool, items, and the information obtained from the tool from the initiation to the end of the development process. What is measured, how it is measured, and the manner in which people are able to respond to questions all need to be informed through input from people with disabilities. What is measured needs to be *important to* them as well as important *for* them so that measurement informs us of the degree to which HCBS supports people with disabilities to achieve personally defined desired life outcomes. Given the relative lack of person-centered measures, further development of a framework and process for developing such measurement tools is warranted. In the following sections, we will describe the process used by the RTC/OM to develop person-centered measures.

## A person-centered measure development process

The Rehabilitation Research and Training Center on HCBS Outcome Measurement (RTC/OM), funded by the National Institute on Disability, Independent Living, and Rehabilitation Research (NIDILRR) was tasked with developing person-centered HCBS quality and outcome measures. The measures, based on the National Quality Forum's (NQF) HCBS Measurement Outcome Framework, were developed based on input from over 350 stakeholders who took part in a series of national participatory planning and decision-making (PPDM) groups. Groups included persons with a variety of disabilities, family members, HCBS providers, and state program administrators. These groups reviewed the NQF Framework to determine the relative importance of each domain and subdomain in the NQF framework. These importance data were used, in conjunction with a gap analysis of existing measures, to prioritize the development of multiple person-centered measures. The PPDM format allowed stakeholders to weigh the importance of potential measure domains and subdomains, add or subtract from the NQF model and move toward consensus as to which were most important to measure.

Much of what has been discussed thus far has focused on person-centered measurement at a broad measure/instrument level. However, the items of which measures are composed are fundamental to person-centered measurement and unfortunately this aspect of measurement has often been neglected. As noted previously, in order to meet the criteria for person-centeredness an item must: (a) be responded to by the person, (b) solicit from the respondent a preference, desire, want, need or emotional state; and (c) provide a chance for the individual to indicate the degree to which those preferences, needs, etc. are being met. It should also be noted that individual should have the opportunity to either indicate the level of importance they place on the content included in the item and/or have the prospect of creating desired life outcomes of their own if items do not correspond well to Table 2 provides examples of items in several NQF domains that are not person-centered as well as items from RTC/OM measures that meet person-centered criteria.

**Table 2:** Examples of person-centered measurement.

NQF Domain	Not Person-Centered	Person-Centered
Social Connectedness	How many times in the last month have you visited with your family members.	I am able to keep in contact with my friends and family members as much as I want.
Choice & Control	How much control do you have over your daily schedule?	I have the amount of control I want/desire over the supports I receive.
Transportation	Logs of community outings	The transportation I use for my leisure and social activities meets my needs.
Meaningful Activity	Frequency counts of community outings over a specified period (e.g., times shopping, out to eat, etc.).	I take part in social activities that I enjoy as much as I want?

The RTC/OM has used an iterative, multi-phased process for developing measures based on extensive stakeholder feedback. This approach is based not only on the belief that measures need to be person-centered but that they should also have strong evidence of their reliability and validity prior to being used. Following the completion of draft items for each measure concept, a technical expert panel (TEP) consisting of people with disabilities, family members, content and measurement experts in each concept area, and HCBS program administrators was convened to review and rate each item that was part of a measure. Reviewers rated items on four-point scales with respect to their importance to the construct, understandability, utility, and feasibility of administration. When items received low scores, reviewers responded to open-ended questions to provide specific feedback related to that item. TEPs also provided feedback on the appropriateness of the response options for each item with respect to their understandability, completeness, and potential ability to accurately convey the experiences of people with disabilities. TEP ratings and feedback were used to revise and, in some cases, remove or replace items that stakeholders indicated did not adequately measure a concept.

An innovative strategy taken during measure concept development was to design the measures under development to be modular as opposed to intended to be used as an instrument. This will allow users interested in better understanding the outcomes experience by HCBS recipients in specific areas to avoid having to administer an entire interest. In addition, each measure has been developed as consisting of two tiers. Four to five Tier-1 items can be used to provide an overall picture of outcomes within a specific domain or across all domains. Tier-2 items which number from 12 to 20 for each measure provide more detailed information with respect to the outcomes experienced by respondents and are intended to support measure users to collect actionable data.

A second step in the measure development process included extensive cognitive testing (CT) of items using the *Cognitive Aspects of Survey Methodology* (CASM) framework (114, 115) and receiving direct input from individuals with disabilities. This process is necessary to confirm items are understood as intended (116, 117) and response options provide respondents with the opportunity to respond in a manner that accurately reflects their thoughts and feelings (118). This form of stakeholder involvement was essential given the intended use of measures with people with a wide variety of disabilities who receive a variety of HCBS.

Following revisions to items based on the results of cognitive testing, all measures were pilot tested with members of each disability population with which they were intended to be used to determine their reliability (internal consistency, test-retest, and inter-interviewer) and the extent to which they were feasible and usable for their intended purpose. Piloting with a diverse sample of adults with disabilities with varying support needs provided measure developers with a more robust set of data related not only with respect to measures and measure items but information about the extent to which the measures developed were suitable for use with HCBS recipients with a variety of disabilities and support needs. Results of the pilot-testing of thirteen person-centered measures spanning seven domains of the NQF HCBS Outcome Measurement Framework (38) have been extremely encouraging with internal consistency (.63–.94; Mean = .81), test-retest (.72–.99; Mean = .85) and inter-rater (.89–.98; Mean = .92) reliabilities on all but one measure found to be at more than acceptable levels. Administration time indicated that most individuals could complete a full measure in no more than 10–15 min and had little difficulty understanding or responding to items indicating a high degree of feasibility with respect to administration.

At the present time, Center staff are in the midst of a national field study being conducted to gather additional information with respect to the psychometric characteristics of the measures that have been developed. Due to the manner in which measures are intended to be used, recruitment is taking place at the provider organization level with multiple participants being recruited from each organization. Participant recruitment has focused on developing a sample of up to 1,000 HCBS recipients with intellectual and developmental disabilities, physical disabilities, traumatic/acquired brain injury, and both psychiatric and age-related disabilities of vary degrees of severity and with a wide range of support needs. Data is being collected over three points approximately 6-months apart with the goal of being able to provide estimates of the degree to which measures are sensitive to change over time. Given that some people with disabilities across all groups of interest may not be able to effectively communicate their experiences as HCBS recipients, a truncated set of proxy measures is also being tested as part of the study.

A final goal of the national field study centers on developing benchmarks against which to compare the outcomes people without disabilities experience in those areas covered by the National Quality Forum's HCBS Outcome Measurement Framework (38). As part of this effort, a national sample of 400 people without disabilities is being surveyed as to the outcomes they experience with respect to a variety of aspects of self-determination/choice and control, social connectedness, meaningful community activity, employment, and transportation. It is hoped that this data will provide an initial set of outcome benchmarks toward which provider organizations can work in an effort to provide people with disabilities with an enhanced degree of equity with respect to outcomes related to a wide variety of aspects of quality of life.

## Conclusion

In this article, we first reviewed the need for HCBS outcome measurement to move beyond its current focus on enumerating the extent to which people with disabilities achieve a predefined set of outcomes (what is important *for* them). We contend that it is just as important to take into consideration an individual's personal needs, preferences, desires, and context (what is important *to* them). If the overall goal of HCBS is to support people with disabilities to lead the lives they desire within inclusive communities, it is imperative that the field move beyond its current focus toward an approach to measurement that is person-centered. This approach is consistent with the HCBS final settings rule (7) in the U.S. and the basic tenets of the Conference on the Rights of People with Disabilities (30) as well as grounded in a paradigm shift in the field of disability services from a *medical* to a *social-ecological model* of disability. We assert that person-centered measurement, which includes a focus on *both* what is important *for* and *to* people with disabilities, is consistent with the National Quality Forum's Framework for HCBS Outcome Measurement (38) and is a key element to fully understanding the effectiveness (or lack thereof) of the supports provided to people receiving community-based services and the quality of outcomes such individuals experience.

The concept of person-centeredness was next explored with a focus on how programs of measurement can move toward a more person-centered approach. This will require a change in focus in many western cultures from measurement focused on comparing the experiences of a target person to benchmarks or outcomes experienced by others to a measurement system in which the “standard” is based on the service recipient's personally defined preferences or goals—not those that other people or the service system sets for them. In the context of measuring outcomes associated with people who receive HCBS, we contend that for measures to truly be person-centered they must be (a) designed with the intent that they will be responded to by people with disabilities themselves; (b) focus on outcomes that are both *important for* and *important to* the recipients of supports; (c) allow people to accurately articulate what they need, outcomes they desire to experience, and the degree to which they view themselves as making progress toward achieving those outcomes; (d) possess the capacity to be used in a longitudinal manner; and (e) permit the individual to indicate the extent to which specific outcomes are of importance to them. As part of this discussion, Goal Attainment Scaling (GAS) was explored as one, but certainly not the only, method for moving toward more person-centered measurement as it potentially allows individuals with disabilities who are receiving supports to quantify the progress they see themselves as making toward personally desired life outcomes or goals. Although the use of this approach does require one to overcome some challenges, evidence of its reliability and validity when used within the rehabilitation sciences is quite encouraging. In addition, in the approach that we advocate, it is the recipients of HCBS supports who identify the desired life outcomes most important to them which then serve as the basis of measurement thus increasing the likelihood that measures are culturally relevant and appropriate.

Given the current state of HCBS outcome measurement, it is clear to us that a framework for the development and validation of person-centered community-based measures would be useful. As an initial step in this direction, we offer the approach to measure concept development that has been used by the University of Minnesota's Research and Training Center on HCBS Outcome Measurement. This approach which is consistent with the NQF's Framework for HCBS outcome Measurement (38) was initiated on the basis of what people with disabilities themselves indicated was most important to measure. It involved a structured, iterative process of item and measure concept development grounded in existing research and theory with respect to the domains of measurement. The iterative process employed allowed a variety of stakeholder groups including people with a variety of disabilities, family members, content area experts, community support providers, and state program administrators to weigh in on the measures under development. Although the process is not yet complete with measures undergoing national field-testing, the results of extensive cognitive testing, and piloting of the measures are quite promising and suggest that this may be an approach to measure development that has potential utility for much needed future efforts in this area.
