# Corrigendum: A person-centered approach to home and community-based services outcome measurement

**DOI:** 10.3389/fresc.2023.1192058

**Published:** 2023-04-12

**Authors:** Matthew A. Roberts, Brian H. Abery

**Affiliations:** Institute on Community Integration, Rehabilitation Research and Training Center on HCBS Outcome Measurement, University of Minnesota, Minneapolis, United States

**Keywords:** HCBS, outcome measurement, measure development, person-centered analysis/approach, person-reported outcome, home and community-based services, person-centered measurement

A Corrigendum on A person-centered approach to home and community-based services outcome measurement

In the published article, there was an error. The description of the CQL Personal Outcome Measures was inaccurate.

A correction has been made to **The need for new approaches to HCBS outcome measurement**, paragraph three. This paragraph previously stated:

“CQL's Personal Outcome Measures (12) although one of the better developed and validated HCBS Outcome tools is part of a commercially available system of assessment and quality improvement. It has been validated with a much wider variety of people with disabilities than the NCI and possesses good psychometric properties. However, the instrument is time-consuming with respect to administration (715 items; 12) limiting its feasibility for many providers. In addition, the CQL-POM, as part of a quality improvement package, is quite expensive to use with onsite administration training alone costing $7,000.”

The corrected paragraph appears below:

“CQL's Personal Outcome Measures (12) is one of the better-developed and validated HCBS Outcome tools. It is part of a commercially available system of assessment that can be used to support provider quality improvement efforts. It has been validated with a much wider variety of people with disabilities than the NCI and possesses good psychometric properties. The instrument is administered in a conversational manner to people with disabilities and includes 171 required items. A manual describing administrative procedures and items is accessible online with training available both in-person and online for organizations interested in using this tool.”

The authors apologize for this error and state that this does not change the scientific conclusions of the article in any way. The original article has been updated.

